# Contribution of carbonate weathering to the CO_2_ efflux from temperate forest soils

**DOI:** 10.1007/s10533-015-0097-0

**Published:** 2015-04-14

**Authors:** Andreas Schindlbacher, Werner Borken, Ika Djukic, Christian Brandstätter, Christoph Spötl, Wolfgang Wanek

**Affiliations:** Department of Forest Ecology, Federal Research and Training Centre for Forests, Natural Hazards and Landscape – BFW, Seckendorff-GudentWeg 8, 1131 Vienna, Austria; Department of Soil Ecology, University of Bayreuth, Bayreuth, Germany; Institute of Geology, University of Innsbruck, Innsbruck, Austria; Department of Microbiology and Ecosystem Science, Faculty of Life Sciences, University of Vienna, Vienna, Austria

**Keywords:** Soil respiration, Carbonate weathering, 13C, Temperate forest

## Abstract

Temperate forests provide favorable conditions for carbonate bedrock weathering as the soil CO_2_ partial pressure is high and soil water is regularly available. As a result of weathering, abiotic CO_2_ can be released and contribute to the soil CO_2_ efflux. We used the distinct isotopic signature of the abiotic CO_2_ to estimate its contribution to the total soil CO_2_ efflux. Soil cores were sampled from forests on dolomite and limestone and were incubated under the exclusion of atmospheric CO_2_. Efflux and isotopic signatures of CO_2_ were repeatedly measured of cores containing the whole mineral soil and bedrock material (heterotrophic respiration + CO_2_ from weathering) and of cores containing only the mineral top-soil layer (A-horizon; heterotrophic respiration). An aliquot of the cores were let dry out during incubation to assess effects of soil moisture. Although the δ^13^C values of the CO_2_ efflux from the dolomite soil cores were within a narrow range (A-horizon −26.2 ± 0.1 ‰; whole soil profile wet −25.8 ± 0.1 ‰; whole soil profile dry −25.5 ± 0.1 ‰) the CO_2_ efflux from the separated A-horizons was significantly depleted in ^13^C when compared to the whole soil profiles (p = 0.015). The abiotic contribution to the total CO_2_ efflux from the dolomite soil cores was 2.0 ± 0.5 % under wet and 3.4 ± 0.5 % under dry conditions. No abiotic CO_2_ efflux was traceable from the limestone soil cores. An overall low contribution of CO_2_ from weathering was affirmed by the amount and ^13^C signature of the leached dissolved inorganic carbon (DIC) and the radiocarbon signature of the soil CO_2_ efflux in the field. Together, our data point towards no more than 1–2 % contribution of abiotic CO_2_ to the growing season soil CO_2_ efflux in the field.

**Electronic supplementary material:**

The online version of this article (doi:10.1007/s10533-015-0097-0) contains supplementary material, which is available to authorized users.

## Introduction

The CO_2_ efflux from forest soils is a major component of the global C cycle. It primarily consists of two biological components, i.e. heterotrophic respiration from decomposers and autotrophic respiration from plant roots and interacting rhizosphere microorganisms (Högberg et al. 2001). Aside these biological sources, a minor abiotic fraction of the total soil CO_2_ efflux can be released during carbonate weathering and subsequent outgassing from soil water. Because weathering of carbonate bedrock proceeds at comparably low rates and because most of the released C is considered to be leached out of the soil, the abiotic component of the soil CO_2_ efflux is generally presumed as marginal (Kuzyakov 2006). Accordingly, the abiotic component of the soil CO_2_ efflux is generally neglected in partitioning approaches and forest C budgeting (e.g. Davidson et al. 2002; Giardina and Ryan 2002; Reichstein et al. 2005). A growing number of studies, however, report high abiotic contributions (10–60 %) to the overall CO_2_ efflux from arable and natural soils in different environments (Čatera and Ogrinc 2011; Emmerich 2003; Inglima et al. 2009; Kowalski et al. 2008; Plestenjak et al. 2012; Ramnarine et al. 2012; Serrano-Ortiz et al. 2010; Stevenson and Verburg 2006; Tamir et al. 2011). Considering that carbonate rock outcrops cover approximately 15 % of the total continental surface area (Amiotte Suchet et al. 2003; Meybeck 1987), an accurate estimate of the soil CO_2_ efflux associated with carbonate weathering is a prerequisite for the understanding and quantification of ecosystem C dynamics in these regions.

Carbonate weathering is predominately controlled by water availability and CO_2_ partial pressure in the soil. Therefore, weathering rates and the contribution of carbonate weathering to the soil CO_2_ efflux will vary with ecosystem productivity, climate, as well as soil and bedrock properties. The succession of wet and dry periods can cause significant CO_2_ uptake and release due to carbonate dissolution and precipitation in arid and semi-arid environments (reviewed in Serrano-Ortiz et al. 2010) whereas carbonate precipitation plays a negligible role in the humid temperate zone. The type of carbonate bedrock (dolomite vs. limestone) influences the production of abiotic CO_2_ as the dissolution rates and weathering intensity are lower for dolomite (Chou et al. 1989; Morse and Arvidson 2002; Pokrovsky et al. 2005) and also vary with morphology and microbial colonization (Davis et al. 2007). Carbonate dissolution based on CO_2_ dissolution and formation and dissociation of carbonic acid is commonly considered as a net CO_2_ sink, and can be expressed as:

1 $${\text{CaCO}}_{3} \left( {\text{calcite}} \right) + {\text{CO}}_{2} + {\text{H}}_{2} {\text{O}} \to {\text{Ca}}^{2 + } + 2{\text{HCO}}_{3}^{ - }$$

2 $${\text{CaMg}}\left( {{\text{CO}}_{3} } \right)_{2} \left( {\text{dolomite}} \right) + 2{\text{CO}}_{2} + 2{\text{H}}_{2} {\text{O}} \to {\text{Ca}}^{2 + } + {\text{Mg}}^{2 + } + 4{\text{HCO}}_{3}^{ - }$$

**Fig. 1:**
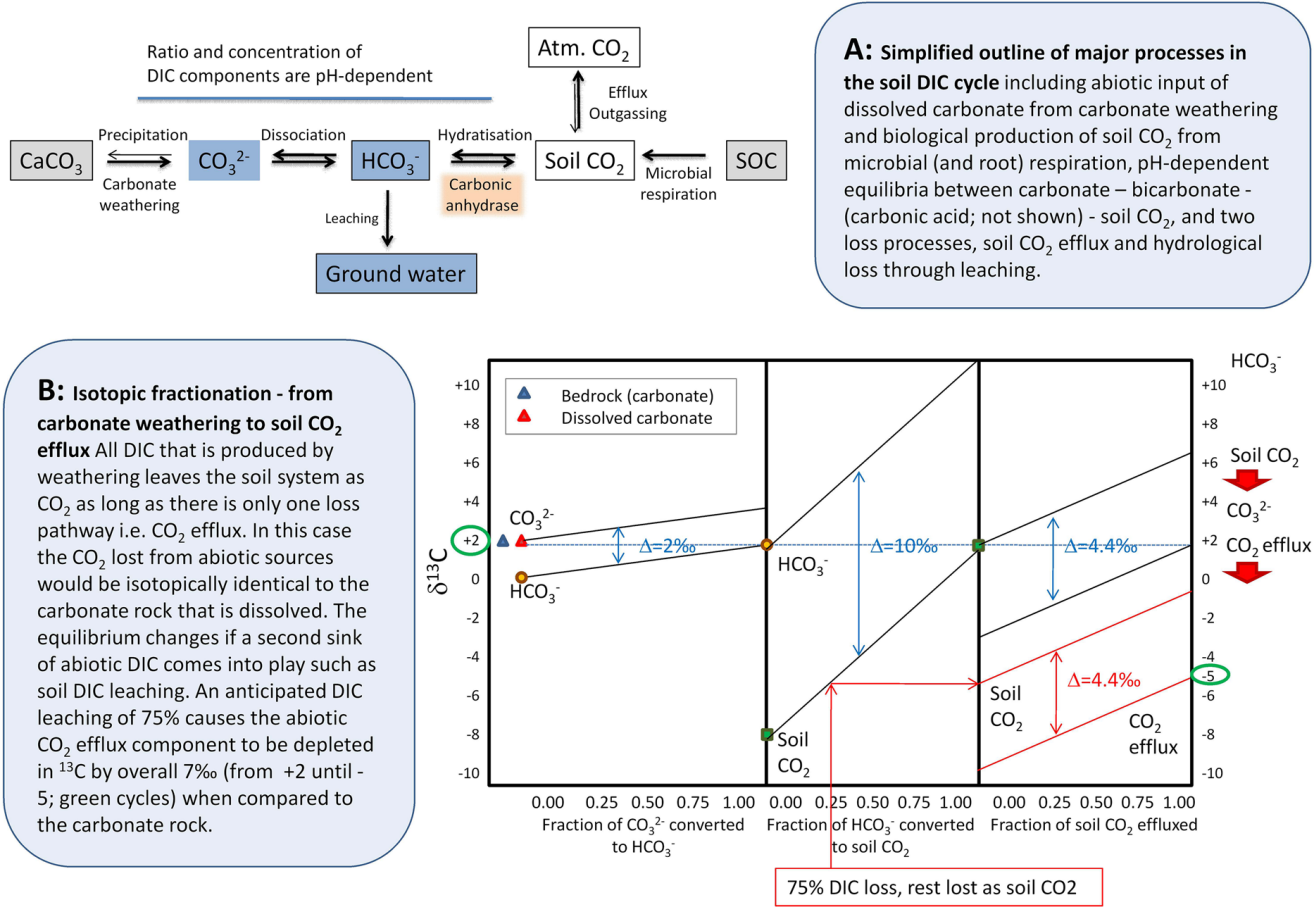
Simplified scheme of the soil DIC cycle (**a**) and conceptual overview of isotope fractionation from carbonate rock to abiotic soil CO_2_ efflux (**b**)

In more temperate humid regions, the majority of the end products of carbonate weathering, the base cations (Ca^2+^, Mg^2+^) and the dissolved inorganic carbon (DIC), are transported from the soils into ground waters and rivers (Fig. 1a) (Ciais et al. 2008; Szramek et al. 2007). Because CO_2_ is consumed during carbonate dissolution, carbonate weathering can become a significant temporal sink of atmospheric or biogenic soil CO_2_ on regional and global scales (Beaulieu et al. 2012; Gombert 2002; Liu and Zhao 1999). Abiotic CO_2_ release due to carbonate precipitation (the reverse reaction of Eq. 1) is less significant in temperate forest soils because soil water is mostly in contact with soil surfaces, carbonate minerals, and soil air. Under specific conditions, however, variations in soil CO_2_ partial pressure, moisture, temperature, or pH can shift the equilibrium conditions towards degassing of CO_2_ and can thereby generate a transient abiotic soil CO_2_ efflux component (Fig. 1a). This abiotic efflux can consist of atmospheric CO_2_ which had entered the soil in the liquid phase already (atmospheric CO_2_ diluted in rainfall) and/or of CO_2_ from carbonate dissolution products.

A number of pH dependent exchange reactions determine the DIC equilibrium in the soil solution (Fig. 1a). In temperate forests, the production and release of organic acids by plant roots and microbes (Attiwill and Adams 1993; van Hees et al. 2005) or the proton input by nitrification, oxidation of organic sulfur and acid rain can foster the dissolution of carbonate and CO_2_ degassing from the soil solution. Enzymes such as carbonic anhydrase which catalyze the conversion between CO_2_ and HCO_3_^−^ in soil solution (Fig. 1a) can positively affect carbonate dissolution rates and abiotic CO_2_ release from the soil solution (Liu et al. 2005; Wingate et al. 2009). Considering the overall rates of geochemical weathering and the climatic preconditions in the temperate zone, the abiotic contribution to the total soil CO_2_ efflux should nevertheless be small (Serrano-Ortiz et al. 2010). However, reliable quantitative assessments of the abiotic CO_2_ efflux component from temperate forest soil are rare.

The CO_2_ efflux from forest soil on carbonate bedrock consists of the following components which differ in their isotopic signature: (I) heterotrophic respiration, (II) autotrophic respiration, (III) abiotic CO_2_ from weathering, and (IV) atmospheric CO_2_ which entered the soil by convection, diffusion or rainwater. The isotopic signature of the heterotrophic respiration is in the range of that of the SOM which is decomposed (δ^13^C between ~−24 and −30 ‰ for C3 plants) but can deviate by several per mill due to the preferential mineralization of specific substrates (Formánek and Ambus 2004; Werth and Kuzyakov 2010). The isotopic signature of autotrophic respiration is in a similar range but can vary for instance with weather conditions which affect the isotope discrimination during photosynthesis (Ekblad and Högberg 2001). The carbonate source material has a distinct isotopic signature with δ^13^C values close to zero whereas the δ^13^C of atmospheric air is close to −8 ‰. Due to its strong signal, the abiotic flux component from carbonate weathering influences the isotopic signature of the total soil CO_2_ efflux even at low contribution. In this study, we use the distinct carbon isotopic signal of abiotic CO_2_ to estimate its contribution to the total soil CO_2_ efflux. As the overall field soil CO_2_ efflux consists of four components, partitioning becomes complex and quantification of a minor component such as the abiotic efflux is hardly feasible. In order to constrain the number of potential CO_2_ sources, we incubated soil cores without plants under the exclusion of atmospheric CO_2_ in the laboratory. Intact soil cores were collected in forests on dolomite and limestone bedrock. We measured CO_2_ and its isotopic signature from cores containing solely the litter and upper mineral soil layer (heterotrophic respiration) and from cores containing the whole soil profiles plus bedrock material (heterotrophic respiration + abiotic CO_2_ from weathering). We hypothesized that (I) the contribution of abiotic CO_2_ would be low because carbonate dissolution is a comparatively slow process and because most of the abiotic C would be leached out of the soil. To assess the effects of soil moisture on abiotic CO_2_ release, we let half of the dolomite soil cores dry out during incubation while soil moisture in the other cores was held at field capacity. We hypothesized (II) that the relative contribution of abiotic CO_2_ to the soil CO_2_ efflux would be higher under the wet treatment because of higher abundance of dissociated carbonic acid in the soil. We further hypothesized that (III) the contribution of abiotic CO_2_ is higher in the limestone soil because calcite dissolution proceeds faster than dolomite dissolution (Chou et al. 1989; Liu et al. 2005; Morse and Arvidson 2002; Pokrovsky et al. 2005). In a parallel experiment we measured soil CO_2_ concentrations and soil CO_2_ efflux as well as their isotopic signature at the dolomite field site throughout the seasons in 2012/2013.

## Materials and methods

### Site description

The dolomite site was located at 910 m a.s.l. on a north–north-east slope of a mountain in the Northern Limestone Alps, close to Achenkirch, Austria (47°34′ 50″N; 11°38′ 21″E). Mean annual air temperature and precipitation were 5.7 °C and 1480 mm (1987–2007), respectively. The 125 year old forest was dominated by Norway spruce (*Picea abies*), with interspersed European beech (*Fagus sylvatica*) and silver fir (*Abies alba*). The soils were a mosaic of shallow Chromic Cambisols and Rendzic Leptosols (FAO 1998). The bedrock was composed of dolomite (Upper Triassic Hauptdolomit Formation). Mull was the dominant humus form with an average thickness of 1–3 cm. A-horizons showed a strong, small-scale variability in thickness reaching from 10 cm up to 40 cm. The C-horizon consists of fine-grained, angular dolomite gravel and reached down (20–40 cm) to the solid bedrock. Between the A and C-horizons a 5–10 cm-thick transitional A/C-horizon was characterised by a mixture of mineral soil and dolomite gravel. Root density was highest in the O and A-horizons and few roots were found down to a depth of 60 cm. Organic C stocks were estimated to be ~10 t ha^−1^ in the organic layer and ~120 t ha^−1^ in the mineral soil (Schindlbacher et al. 2010).

The limestone site was located on a south–south-west slope of the Hochschwab massif in the Northern Limestone Alps, Austria (47°34′02″N; 15°02′19″E). Mean annual air temperature was between 4 and 5 °C. Mean annual precipitation was 1450 mm. The dominant tree species in the montane region (800–1400 m) were Norway spruce and European larch (*Piceaabies* and *Larix decidua*). The soils were LepticHistosols (FAO 1998) formed on limestone (Middle Triassic Wettersteinkalk Formation). The O-horizon depth at the sampling site was 1–4 cm. The A-horizons depth varied between 10 and 20 cm. As at the dolomite site, the C-horizon material consisted of fine gravel (20–50 cm deep).

### Soil column sampling and treatment

At the dolomite site, soil was sampled at five randomly distributed locations in late November 2011. From each location a pair of columns containing whole soil profile and an additional column containing only the A-horizon was sampled for incubation. Sampling was performed as little destructive as possible. A Plexiglass cylinder (20 cm diameter × 60 cm length for whole soil profiles; 20 cm diameter × 30 cm length for A-horizons) was pushed into the soil after cutting the roots around the cylinder edge with a knife. This procedure worked well until larger stones in the C-horizon blocked the insertion of the cylinder. Cylinders containing the undisturbed soil were then taken out and the last part of the C-horizon was filled from below with a shovel. Five cores with whole soil profiles as well as five A-horizon cores were watered to field capacity. No water was added to the remaining five cores with whole soil profiles. Accordingly, at the dolomite site, three different sets of cores were incubated; “wet” (whole profile), “dry” (whole profile) and the separated “A-horizon” with a replication of five columns each.

After the dolomite soil incubation was finished, we sampled (same procedure) cores from four randomly distributed locations at the limestone site. We took four whole-profile cores and four cores containing only A-horizons in mid-October 2012. Cores from the limestone site were incubated at the original water content and the corresponding set of cores for limestone soil were “whole profile” and “A-horizon” with a replication of four cores each. Soil moisture of the limestone cores was kept constant at the original water content by periodical watering as described above until day 45 of the incubation. At day 45, soil moisture of all cores from the limestone site was increased to field capacity and kept at this level until the end of incubation.

### Incubation and sampling

**Fig. 2:**
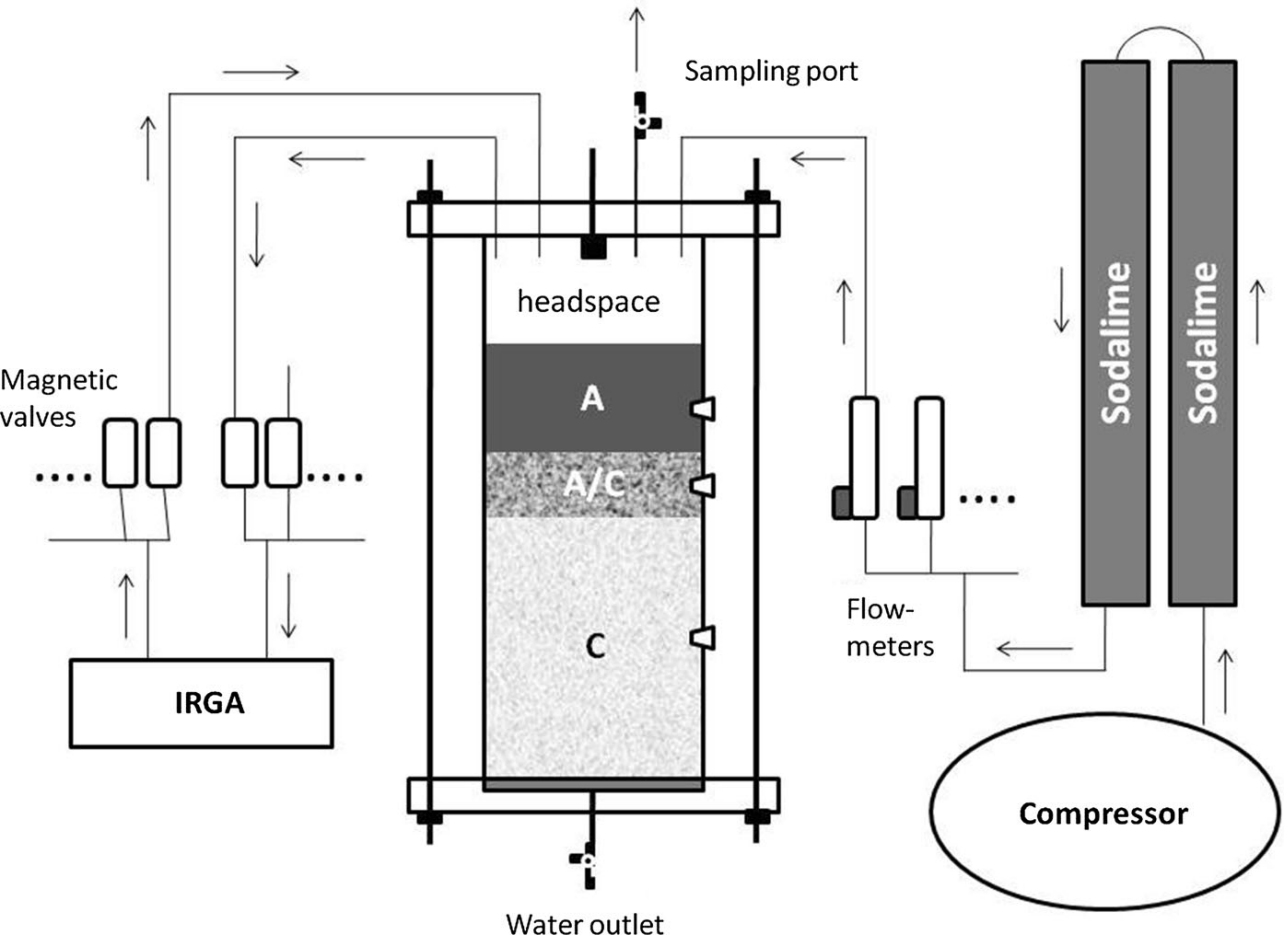
Schematic drawing of the incubation system (*arrows* indicate the direction of air-flow). Ambient air was compressed and pumped through sodalime-columns to scrub ambient CO_2_. Flow rates to the soil column headspace were regulated in a way that headspace CO_2_ concentrations ranged between 380 and 400 ppm. The flushing-air left the soil column headspace through an outlet which was also used as sampling port for isotopic analyses. Two benches of magnetic valves (*inlet*, *outlet*) allowed to switch between individual soil columns (n = 15) for CO_2_ concentration measurements with an IRGA. Water was added through a spray valve at the top of the column headspace and leaching water was collected from an outlet at the bottom of the soil column. At each soil horizon, a septum was installed into the column wall to allow direct sampling of soil–air with a syringe

Soil cores were incubated in complete darkness at a temperature of 20 ± 1 °C. For CO_2_ measurements (Fig. 2), soil columns were closed at the top and bottom and attached to the flushing system. In our attempt to expel all atmospheric CO_2_ from the soil columns, CO_2_-free air was pumped through each soil column from top to bottom at a flow rate of 10 L h^−1^ during the first week. After a week the bottom exhaust was closed and only the headspace of the soil column was flushed for further 2 weeks (acclimation period). Using adjustable flow meters, the flow of CO_2_-free air through the headspace (~10–15 cm height) of each soil column was regulated manually to a rate at which the column CO_2_ headspace concentration stabilized at 380–400 ppm. The CO_2_-free air was produced from ambient air which was compressed and blown through two consecutive columns (12 × 100 cm each) filled with sodalime granulate.

CO_2_ concentrations in the soil-column headspaces were measured with an infrared gas analyzer (IRGA) (EGM-4, PP-Systems, Amesbury). A control unit of 30 magnetic valves allowed switching between column headspaces for CO_2_ concentration measurements in a completely closed system (Fig. 2). For the determination of CO_2_ efflux rates, the headspace-flush was interrupted and after a 1 min equilibration time the CO_2_ concentration in the chamber headspace was measured every 30 s throughout 3 min. The CO_2_ efflux was derived from the linear concentration increase over time.

CO_2_ concentrations of soil air were measured directly from septa installed in the column wall at three depths (Fig. 2). A short (~20 cm) tubing (inner diameter 4 mm) was attached to the inlet of a second IRGA. The tubing ended in a 5 cm-long syringe needle which was directly inserted into the septa/soil. After ~5 s, the IRGA showed a steady value of the soil–air CO_2_ concentration.

Air samples for isotopic analyses were obtained from both, the soil column headspace and from the three soil horizons of each core. The headspace sampling port which simultaneously served as the outlet of the flushing air was equipped with a stopcock and a three-way Luer lock. For sampling, a syringe (25 mL) was attached and the stopcock and needle volume were flushed with headspace air by using the Luer lock. For isotopic analysis, 12 mL of headspace air were injected into 12 mL glass vials (Exetainer, Labco Ltd, High Wycombe, UK) containing CO_2_-free air. While injecting the sample, we inserted a second needle to allow outflow of air in order to avoid overpressurizing the vials. The second needle was removed shortly before the full sample was injected thereby leaving a minimal overpressure in the vial. Soil air samples from the soil horizons were obtained through three septa which were installed into the column wall. A syringe needle was inserted directly into the soil and 6 mL of soil air were sampled with a 25 mL syringe. Before sampling, the syringe needle space was flushed with another 4 mL of soil air as described above with a Luer lock system. Soil air was injected into a 12 mL vial as described above for the headspace sampling.

The carbon isotope ratios of the CO_2_ were then analyzed by continuous-flow isotope-ratio mass spectrometry (IRMS) on a Delta V Advantage Mass Spectrometer coupled to a GasBench system (Thermo Fisher Scientific, Bremen, Germany). CO_2_ efflux and isotopic signatures were determined every 2–3 weeks throughout 166 days incubation of the dolomite soil and 60 days incubation of the limestone soil.

We also collected drainage water to estimate the flux and isotopic ratio of dissolved inorganic carbon (DIC). This analysis was restricted to the dolomite soil and to the “wet” and “A-horizon” cores. We simulated rainfall events by slowly adding larger quantities (400 mL for whole profiles; 200 mL for A-horizons) of water, equivalent to ~12 and ~6 mm of rainfall. Drainage water was collected 4 h after irrigation from the bottom outlet of the column. We collected 5 mL with a syringe and pressed 3 mL through a Teflon filter (0.45 µm mesh size) attached to the syringe. The first 2 mL were used to flush the needle; the third ml of filtered soil water was injected into an evacuated 12 mL vial containing ~1 µL concentrated phosphoric acid. Concentrations and isotopic signatures of the evolving CO_2_ were measured as described above by GasBench-IRMS. DIC was sampled less frequently than CO_2_ with in total four sampling dates throughout the 166 day incubation.

### Soil and bedrock analysis

After incubation, soil columns were disaggregated and the dry weight, stone content, water content, pH, carbonate content, contents of organic C (C_org_) and total N as well as the isotopic signature of the C_org_ of each soil horizon were determined. Dry weight and gravimetric water content were determined after drying ~50 g of sieved soil at 105 °C for 12 h. Volumetric stone content was estimated by dividing the horizon specific mass of stones larger than 2 mm by the density of dolomite (2.9 g cm^−3^) and limestone (2.7 g cm^−3^) respectively. Soil pH was measured potentiometrically according to ISO 10390 (www.iso.ch). For determination of the carbonate content, ground soil samples were treated with a strong acid (10 % HCl). The volume of the carbon dioxide produced was measured by using a calcimeter (Scheibler unit), and was compared with the volume of carbon dioxide produced by pure carbonate (ISO 10693; www.iso.ch). Total C and N contents of the soil horizons were determined with a LECO CN-2000 dry combustion analyzer (www.leco.org). Organic C content was assessed by correcting total soil C by carbonate contents (ISO 10694; www.iso.ch). The isotopic signature of C_org_ from the different soil horizons was determined after decarbonatization with a Flash EA elemental analyzer coupled via ConFlo III interface to a Delta^Plus^ IRMS system (Thermo Fisher Scientific, Bremen, Germany). For pre-treatment aliquots of finely ground soil (100 mg) were treated with 1 mL 2 M HCl at room temperature until the full decarbonisation of the samples and subsequently dried in a drying oven at 60° C for 2 days.

Dolomite and calcite of the carbonate bedrock material were reacted with phosphoric acid at 90 and 72 °C, respectively, and analysed using an automated continuous-flow Delta^Plus^XL isotope ratio mass spectrometer at the University of Innsbruck. Calibration of dolomite samples was accomplished using a dolomite standard, whose isotopic composition was previously determined using classical offline preparation (provided by T. Vennemann, Lausanne). Calibration of calcite samples was based on NBS19, CO1 and CO8 reference materials. Results are reported with respect to the VPDB scale, and the long-term analytical uncertainties at the 1σ level is equal 0.07 for δ^13^C (Spötl&Vennemann Spötl and Vennemann 2003).

### Field measurements

Soil CO_2_ efflux, soil air CO_2_ concentrations and the corresponding isotopic signatures were assessed in the field at the dolomite site in 2012/2013. Measurements were performed once during spring (16 May), summer (09 July), autumn (08 October), and winter (26 February). Three soil pits were equipped with stainless steel capillary tubes (inner diameter 1 mm) attached to perforated 4 cm-long Teflon tubes (inner diameter 4 mm, inserted into the side walls of the soil pits) to assess the CO_2_ concentrations within the soil profiles. Capillary tubes were installed in the A-horizon, the A/C-horizon and the C-horizon, and the pits thereafter carefully filled with horizon-specific soil material. Soil CO_2_ concentrations were assessed by directly connecting the IRGA to the steel capillaries. Samples for isotopic analyses were taken with a syringe and transferred into 12 mL vials as described above. Surface soil CO_2_ efflux during the snow-free season was estimated from closed dynamic chamber measurements as described by Schindlbacher et al. (2009) (one chamber per soil profile) and by a CO_2_ concentration gradient method applied during winter (Schindlbacher et al. 2014). To assess the isotopicsignature of soil respired CO_2_ during the snow free season, the Keeling plotapproach was used (Keeling 1958). The interceptof a linear regression of δ^13^C of sampled CO_2_versus 1/[CO_2_] provided an estimate of δ^13^C ofsoil-respired CO_2_ (where [CO_2_] was the CO_2_ concentrationin %). During snow cover, the isotopic composition of the soil CO_2_ efflux was estimated from the δ^13^C along the CO_2_ gradient in the snow cover according to Bowling et al. (2009) and Davidson (1995).

### Data analysis and estimate of the abiotic CO_2_ efflux

Treatment effects on isotopic signatures of the soil CO_2_ efflux and soil CO_2_ concentration were statistically tested by one-way repeated measures ANOVA (procedure GLM, SAS Institute Inc., Cary, NC, USA). The fractional contribution of CO_2_ derived from weathering (*f*) to overall soil CO_2_ efflux from the incubated soil columns was calculated following the two-pool mixing model:

3 $$f = \frac{{\delta^{13} C_{total} - \delta^{13} C_{microbial} }}{{\delta^{13} C_{carbonate} - \delta^{13} C_{microbial} }}$$

where *δ*^*13*^*C*_*total*_ is the C isotope signature (‰) of the CO_2_ efflux from the whole soil profile, *δ*^*13*^*C*_*microbial*_ is the C isotope signature (‰) of the CO_2_ efflux from the separately incubated A-horizons, and *δ*^*13*^*C*_*carbonate*_ is the C isotope signature (‰) of CO_2_ originating from carbonate (dolomite, limestone) weathering. The mixing model applied assumes that the abiotic contribution to the CO_2_ efflux from A-horizon cores is zero or negligible. This assumption was challenged as some of the A-horizon cores contained up to 200 mg carbonate g^−1^ dw (see results; Table 1). Nonetheless, we found strong evidence that the CO_2_ efflux from the A-horizon cores contained no or only negligible amounts of abiotic CO_2_. There was no relationship between the δ^13^C of the CO_2_ efflux and the carbonate content of the separately incubated A-horizons (Fig. S1) ranging from 0 to 200 mg g^−1^ dry weight. Furthermore, the absolute amount of carbonate in the upper layer of the mineral soil (A-horizon) was almost two orders lower when compared to the amount of carbonate in the deeper soil layers. Therefore, the insignificant contribution of abiotic CO_2_ to the soil CO_2_ efflux of the A-horizon cores was neglected in our mixing model. Alternatively, we run the same calculations with the isotope signatures of soil organic matter (C_org_, weighted for the whole profile) as proxy for *δ*^*13*^*C*_*microbial*_. The results that we obtained that way were very similar and in some cases contributions from carbonate weathering were even lower (data not shown).

**Table 1:** Properties of dolomite (mean ± SE; n = 5) and limestone soil cores (mean ± SE; n = 4) and the corresponding separately incubated A-horizons. All parameters were assessed after disaggregation of the columns after finishing the incubation

Bedrock	Dolomite				Limestone	
	Hori-zon	Whole profile wet	Whole profile dry	A-horizon	Whole-profile	A-horizon
Depth (cm)	FF	1.1 (0.4)	1.5 (0.4)	1.0 (0.2)	1.9 (0.3)	0.40 (0.1)
	A	13.1 (1.1)	12.9 (0.8)	6.3 (0.4)	10.9 (1.5)	10.8 (1.3)
	A/C	6.5 (6.5)	5.9 (0.5)		7.9 (1.8)	
	C	16.8 (1.1)	16.1 (1.5)		15.8 (1.8)	
Dry weight (g) forest floor and soil <2 mm	FF	20 (9)	41 (13)	29 (5)	18 (4)	18 (4)
	A	910 (132)	1024 (213)	363 (36)	508 (81)	533 (202)
	A/C	578 (66)	623 (30)		2207 (1016)	
	C	1508 (136)	1338 (288)		4777 (589)	
Stones >2 mm (vol%)	FF					
	A	6.6 (2.5)	4.2 (1.6)	0.7 (0.3)	0.9 (0.3)	1.1 (0.5)
	A/C	23.7 (2.4)	23.8 (6.0)		18.2 (3.6)	
	C	49.5 (2.8)	49.7 (2.7)		50.8 (13.8)	
Water content (mass%; post-incubation)	FF	62.7 (3.2)	33.1 (4.9)	72.6 (1.5)	72.7 (0.9)	74.5 (4.3)
	A	61.9 (2.2)	48.5 (4.1)	65.7 (1.9)	70.6 (1.8)	70.6 (3.0)
	A/C	42.2 (2.4)	36.3 (1.9)		35.3 (6.9)	
	C	21.5 (3.6)	15.0 (3.0)		25.6 (2.2)	
pH	FF	5.9 (0.4)	5.8 (0.3)	5.5 (0.4)	5.8 (0.5)	5.9 (0.4)
	A	6.8 (0.2)	6.7 (0.3)	6.9 (0.1)	6.4 (0.5)	6.2 (0.5)
	A/C	7.3 (0.0)	7.2 (0.1)		7.1 (0.1)	
	C	7.4 (0.1)	7.6 (0.1)		7.4 (0.0)	
CaMg(CO_3_)_2_ (dolomite) CaCO_3_ (limestone) (mg g^−1^ dw)	FF					
	A	105 (18)	84 (28)	121 (44)	197 (81)	173 (91)
	A/C	528 (50)	414 (97)		722 (48)	
	C	817 (54)	821 (39)		780 (68)	
Corg (mg g^−1^ dw)	FF	344 (15)	293 (40)	367 (14)	413 (23)	398 (31)
	A	172 (23)	177 (13)	162 (11)	272 (40)	296 (42)
	A/C	61 (8)	72 (5)		58 (11)	
	C	21 (3)	22 (4)		24 (5)	
Isotopic signature Corg (δ^13^C ‰)	FF	−28.21 (0.37)	−28.60 (0.27)	−28.85 (0.36)	−28.46 (0.11)	−28.42 (0.09)
	A	−26.30 (0.16)	−26.26 (0.02)	−26.38 (0.06)	−27.08 (0.17)	−27.28 (0.20)
	A/C	−24.97 (0.19)	−25.34 (0.22)		−25.60 (0.37)	
	C	−24.06 (0.40)	−24.44 (1.24)		−25.20 (0.71)	
Bedrock C (δ^13^C ‰)						
CaMg(CO_3_)_2_ (Dolomite)		+2.92 (0.04)	(0.04)			
CaCO_3_ (Limestone)					+2.12 (0.04)	
N tot (mg g^−1^ dw)	FF	16.3 (0.6)	15.2 (1.7)	15.9 (1.0)	22.3 (0.6)	21.4 (1.0)
	A	11.1 (1.1)	11.7 (1.1)	10.5 (0.7)	17.2 (2.3)	18.5 (0.7)
	A/C	4.4 (0.6)	5.0 (0.4)		3.3 (0.9)	
	C	0.7 (0.3)	0.8 (0.3)		0.6 (0.1)	

The isotopic signature of the CO_2_ from carbonate weathering (*δ*^*13*^C_*carbonate*_) was not directly measured but estimated from isotopic measurements of bedrock carbonate (dolomite, limestone). To account for potential isotope fractionation during the weathering process and during the transformation from HCO_3_^−^ to gaseous CO_2_ we assumed steady state conditions in an open system (isotopic equilibrium conditions) during our soil CO_2_ measurements (Fig. 1b). Under such (e.g. in well-drained soils) there is a C isotope equilibrium effect between Ca carbonate and bicarbonate (~1–2 ‰ ^13^C depletion of bicarbonate relative to Ca carbonate) and between bicarbonate and soil CO_2_ (~10 ‰ ^13^C depletion of soil CO_2_ relative to bicarbonate, Fig. 1b) (Amundson et al. 1998; Cerling 1984; Emrich and Vogel 1970; Halas et al. 1997; Mook et al. 1974; Nordt et al. 1998). These equilibrium isotope effects are additive and slightly temperature-dependent, i.e. the lower the temperature the larger the equilibrium isotope effect (Halas et al. 1997; Myrttinen et al. 2012). Moreover, soil CO_2_ is enriched by ^13^C by up to ~4 ‰ relative to soil CO_2_ efflux due to diffusional isotope fractionation during CO_2_ escape from the soil (Cerling et al. 1991), but this fractionation is lower when CO_2_ efflux is triggered by advective instead of diffusive soil gas transport (Kayler et al. 2010). In an open system the fraction of weathered (i.e. dissolved) carbonate that is emitted as CO_2_ from the soil determines whether the effluxed CO_2_ reflects the isotopic composition of the carbonate or not (Hendy 1971). We would expect the same isotopic signature of both the carbonate bedrock and soil abiotic CO_2_ efflux if all C released through weathering is emitted from soils in the form of CO_2_ (Fig. 1b). If a larger fraction of DIC is lost through hydrological pathways (e.g. leaching of 75 % of the bicarbonate produced and only 25 % is converted to soil CO_2_), as was anticipated for the studied forest soil, then soil CO_2_ should be ^13^C-depleted (by approximately −5 ‰) relative to carbonate (+2 ‰), resulting in an isotopic offset of about −7 ‰ relative to carbonate rock which was taken into account in the isotopic mixing model (Eq. 3). In some cases, e.g. glacial settings, carbonate weathering does not occur under steady state (equilibrium) conditions. In this case kinetic isotope fractionation with up to 17 ‰ enrichment in ^13^C (Skidmore et al. 2004) may occur which causes DIC and soil CO_2_ efflux to become intermittently ^13^C-depleted relative to the carbonate bedrock, until equilibrium conditions are reached. Kinetic isotope fractionation during carbonate dissolution may be expected to occur when soil water-carbonate contact times are short, e.g. shortly after rainfall events but can be excluded in our experimental setup.

## Results

### Soil properties

Carbonate contents sharply increased from litter (zero) to ~800 mg g^−1^ in the C-horizon soil fraction. The stone content increased with depth and was roughly 50 % of the C-horizon volume in the incubated cores (Table 1). The carbonate content of the separately incubated A-horizons showed high spatial variability ranging from 10 to 200 mg g^−1^ in the cores from the dolomite site and from 0 to 280 mg g^−1^ in the cores from the limestone site. Isotope signatures (δ^13^C) of C_org_ significantly increased (linear regression; p < 0.0005) with soil depth at both sites from −28 ‰ in the litter layer to −25 ‰ in the C-horizon (Table 1). Bedrock material showed δ^13^C signatures of +2.9 ‰ for dolomite and +2.1 ‰ for limestone.

**Fig. 3:**
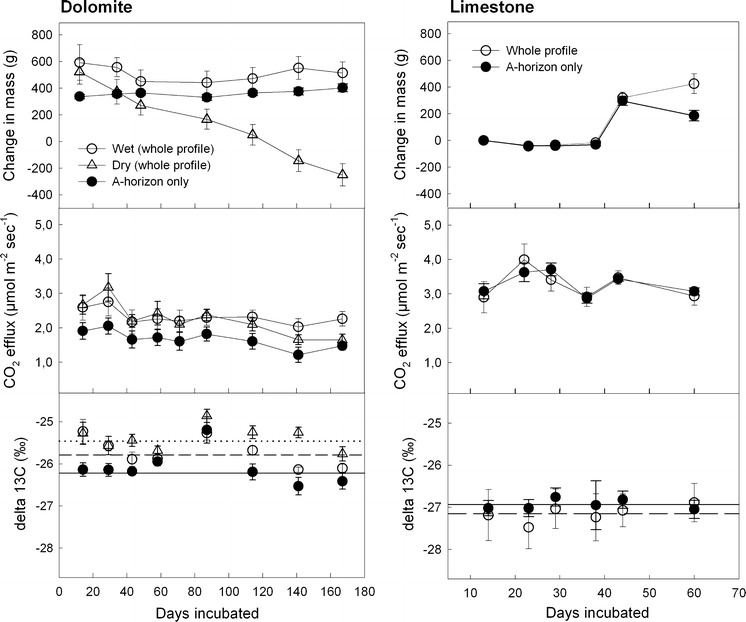
Soil CO_2_ efflux and its isotopic signature from dolomite (*left panel*) and limestone (*right panel*) cores (mean ± SE; Dolomite n = 5; Limestone n = 4). Temporal changes in soil-core mass (*upper panel*) reflect changes in soil moisture. A set of complete dolomite-soil profiles was initially watered and incubated at near field capacity (Wet *open circles*) whereas a second set was allowed to dry out (Dry *triangles*). A third set contained solely A-horizons (*full circles*) but no dolomite gravel. Limestone-soil was incubated in sets of whole soil profiles (*open circles*) and A-horizons only (*full circles*) which were all watered at incubation day 45. Lines in the lowermost panel indicate means over all sampling dates except day 86 (Dolomite: Wet *dashed*; Dry *dotted*; A-horizon *full*; Limestone: whole profile *dashed*; A-horizon *full*). At day 86 leaky seals of vial caps likely biased the δ^13^C measurements

Watering to field capacity initially increased the weight of the dolomite soil cores by 400–600 g (Fig. 3). Soil moisture and the weight of the soil cores, were kept constant during the wet treatment, whereas in the dry treatment, the soil cores gradually dried out and lost weight (Fig. 3). At the end of the incubation period soil moisture contents of the organic layers and A-horizons were significantly lower in the dry treatment cores (33 and 48 mass%) than in the wet treatment cores (both 63 mass%) (Table 1).

### Soil CO_2_ efflux and soil CO_2_ concentrations

**Fig. 4:**
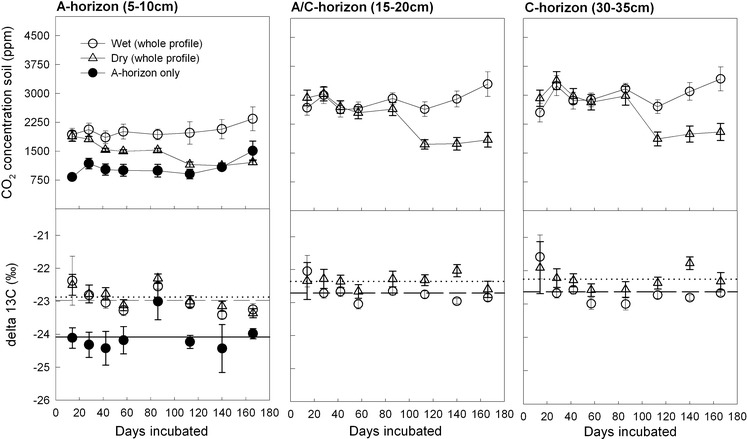
CO_2_ concentrations and isotopic signatures (mean ± SE, n = 5) of soil air collected in the A, A/C, and C horizons of the incubated dolomite soil cores. Centimeter values in *brackets* indicate the depth of the sampling point. For wet (*open circles*) and dry (*triangles*) treatments, complete soil profiles were incubated. A set of A-horizon only cores (*full circles*) was incubated for comparison. Lines in the lower panel indicate means over all sampling days except day 86 when leaky seals biased the δ^13^C measurements (Wet *dashed*; Dry *dotted*; A-horizon *full*)

While the CO_2_ efflux from the limestone soil cores and the separately incubated A-horizons was in a similar range, the CO_2_ efflux of the dolomite soil cores was continuously higher than that of the separately incubated A-horizons (Fig. 3). This can be explained by the lower amount of top-soil which was incubated for the dolomite site (Table 1). As the deeper layer of the A-horizon at the dolomite site already contained stones, we only incubated the uppermost layer. The CO_2_ efflux from dolomite soil showed a slightly decreasing trend throughout the 166 day incubation period (Fig. 3). The CO_2_ efflux was similar under wet and dry treatment during the first 86 days of incubation. Dry treatment efflux rates decreased more pronouncedly during the latter part of incubation (day 113–166; Fig. 3). The effect of drying became more evident in soil CO_2_ concentrations which gradually decreased in the A-horizon from the beginning onwards (Fig. 4). CO_2_ concentrations in the deeper soil horizons of the dry treatment remained relatively constant until day 86 of the incubation but dropped significantly afterwards (Fig. 4). Watering of the limestone soil cores did neither affect the CO_2_ efflux from whole limestone soil cores nor from the respective A-horizons.

### Isotopic signature and CO_2_ efflux partitioning

The isotopic signature of soil CO_2_ efflux varied within a narrow range throughout the incubations of both the dolomite and limestone soil (Fig. 3). At day 86, δ^13^C values were unusually high for all sets of cores of the dolomite soil incubation, suggesting influx of atmospheric CO_2_ due to leaky sealing of the Exetainer vials. We therefore rejected this date for statistical analysis of the δ^13^C values of soil CO_2_ efflux and soil CO_2_. Average δ^13^C values throughout the dolomite soil incubation were −26.2 ± 0.1 ‰ for the separately incubated A-horizons, −25.8 ± 0.1 ‰ for the wet soil cores, and −25.5 ± 0.1 ‰ for the dry soil cores (p = 0.015, repeated measures ANOVA). Applying the two-pool mixing model (Eq. 3) we calculated an average abiotic contribution of 2.0 ± 0.5 % to the total soil CO_2_ efflux from the wet dolomite soil cores. The mean abiotic contribution to the dry treatment CO_2_ efflux was 3.4 ± 0.5 % when calculated for the whole incubation period. The estimated abiotic contribution to the total soil CO_2_ efflux was lower during the first phase of drying (until day 57; mean contribution 2.8 ± 0.6 %) than during the phase during which soil moisture was at lowest levels (day 58–166; mean contribution 4.3 ± 0.8 %). δ^13^C values of the CO_2_ efflux from the wet cores decreased with incubation time (linear regression, p < 0.05) whereas δ^13^C from dry cores and the separated A-horizons did not show a clear temporal trend. Generally, soil CO_2_ efflux was 2–3 ‰ depleted when compared with soil air CO_2_, pointing to kinetic isotope fractionation during soil CO_2_ efflux (Figs. 3, 4). The soil CO_2_ in the A-horizons of the whole dolomite soil cores was significantly ^13^C enriched compared to that in separately incubated A-horizons (p = 0.0013, repeated measures ANOVA) (Fig. 4). In dolomite soil cores, the δ^13^C values of soil CO_2_ in the A/C and C-horizons were slightly higher than in the A-horizons (Fig. 4). Moisture treatment (wet, dry) had no significant effect on the isotopic signature of soil CO_2_ in any horizon.

**Fig. 5:**
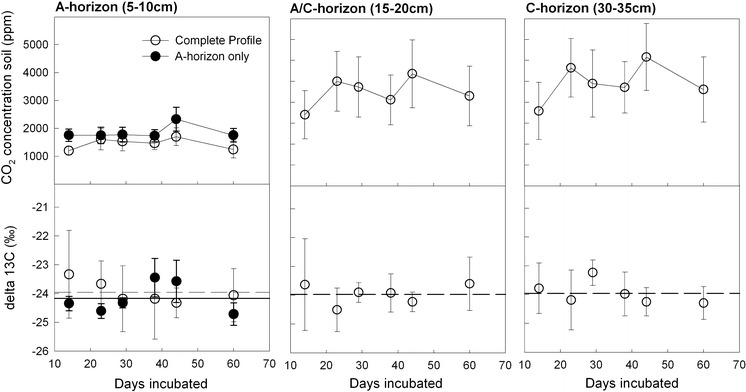
CO_2_ concentrations and isotopic signatures (mean ± SE, n = 4) of soil air sampled from the A, A/C, and C horizons of the incubated limestone soil cores. Centimeter values in *brackets* indicate the depth of the sampling point. Soil was incubated in sets of complete profiles (*open circle*) and A-horizons only (*full circles*). Lines in the lower panel indicate means over all sampling days (Complete soil profile *dashed*; A-horizon *full*)

The isotopic signature of the soil CO_2_ efflux of limestone soil cores showed higher spatial variability when compared to that of the dolomite soil cores (Fig. 3) but the mean δ^13^C signatures of soil CO_2_ efflux were nearly identical for separately incubated A-horizons (−26.9 ± 0.1 ‰) and whole limestone soil cores (−27.2 ± 0.1 ‰). Due to the insignificant (p = 0.63, repeated measures ANOVA) isotopic differences, an abiotic contribution to the total soil CO_2_ efflux of limestone soil cores could not be detected. There was also no clear temporal pattern regarding the isotopic signature of limestone soil CO_2_ efflux throughout the 60 day incubation period. Mean δ^13^C values of CO_2_ in the A-horizons were similar between whole soil cores and separated A-horizons and were also similar to the δ^13^C values of soil CO_2_ in the A/C and C horizons (Fig. 5). Watering of limestone soil cores at day 45 did not affect the isotopic signature of the CO_2_ efflux or the soil CO_2_.

### Dissolved inorganic carbon (DIC)

DIC concentrations in drainage water from whole soil cores were higher (average over all 4 dates: 30.7 ± 0.7 mg L^−1^) than from separated top-soil (14.7 ± 0.9 mg L^−1^) (Table 2). Drainage water DIC from the whole soil profile cores was more ^13^C enriched (−15.2 ± 0.1  ‰) than drainage water from the top-soil cores (−17.7 ± 0.5  ‰) (Table 2).

**Table 2:** Dissolved inorganic carbon (DIC) concentration and isotopic signature in drainage water of dolomite soil cores

Days incubated	DIC (sample ppm CO_2_)		DIC (mg/L)		δ^13^C (‰)	
	Whole profile	A-horizon	Whole profile	A-horizon	Whole profile	A-horizon
43	5613 (230)	2693 (1302)	30.9 (1.3)	14.8 (3.9)	−15.14 (0.58)	−17.99 (0.38)
114	5211 (58)	2288 (1550)	28.7 (0.3)	12.6 (3.6)	−15.09 (0.32)	−17.90 (0.42)
141	5941 (280)	3098 (1715)	32.7 (1.5)	17.4 (8.7)	−14.98 (0.35)	−18.60 (0.54)
167	5540 (297)	2579 (1445)	30.5 (1.6)	14.8 (5.4)	−15.56 (0.38)	−16.20 (0.22)

Drainage water was collected from the wet treatment of whole soil profile cores (Whole profile) and from separately incubated A-horizons 1 h after water addition

### Field measurements

The in situ soil CO_2_ efflux showed the typical seasonal pattern with highest flux rates during summer and lowest flux rates during winter (Table 3). The summertime isotopic signature of the field soil CO_2_ efflux was very similar to that of the soil cores in the laboratory (dolomite cores) (Table 3; Fig. 3). However, the average isotopic signatures of the field soil CO_2_ efflux varied substantially throughout seasons, i.e. between −24.7 ‰ in spring and −27.7 ‰ in winter. Summertime field soil CO_2_ concentrations were in all soil horizons approximately twice as high as in the incubation study (Table 3). The δ^13^Cvalues of soil CO_2_ were close to those in the incubation study. During spring, soil CO_2_ in the A-horizon was most depleted in ^13^C and became more ^13^C enriched with increasing soil depth (Table 3). This pattern reversed during the other seasons during which soil CO_2_ in the A-horizon was most ^13^C enriched and CO_2_ in the C-horizon was most depleted. Wintertime δ^13^C values of soil CO_2_ were generally less negative when compared with those of the warmer seasons.

**Table 3:** Field CO_2_ data (mean ± SE, n = 3) throughout the seasons 2012/13 (spring 16.05.2012, summer 09.07.2012, autumn 08.10.2012, winter 26.02.2013)

Horizon	Spring	Summer	Autumn	Winter
CO_2_ efflux (µmol m^−2^ s^−1^)				
	2.43 (0.07)	4.84 (0.97)	2.33 (0.53)	0.33 (0.04)
CO_2_ efflux (δ ^13^C ‰)				
	−24.68 (0.62)	−25.62 (0.23)	−26.25 (0.08)	−27.71 (0.02)
CO_2_ concentration in soil (ppm)				
A	1431 (249)	4004 (1026)	3319 (288)	1430 (234)
A/C	2439 (479)	6313 (1674)	4986 (399)	1476 (541)
C	3909 (581)	8800 (1439)	7055 (778)	2143 (576)
Isotopic signature of soil CO_2_ (δ ^13^C ‰)				
A	−22.39 (0.54)	−21.86 (0.78)	−21.36 (0.80)	−18.98 (0.77)
A/C	−21.19 (0.97)	−23.02 (0.54)	−22.59 (0.50)	−19.73 (1.21)
C	−19.30 (0.37)	−23.72 (0.31)	−23.16 (0.29)	−20.59 (0.84)

Soil CO_2_ efflux was estimated from closed dynamic chamber measurements during the snow-free season and by a snow-CO_2_ gradient method during winter. Isotopic signature of the CO_2_ efflux were derived from Keeling-plots. Soil CO_2_ concentrations and δ^13^C values were determined from soil air sampled directly out of the soil profile at different depths (A-horizon 8 ± 1 cm; A/C-horizon 20 ± 3 cm; C-horizon 38 ± 4 cm)

## Discussion

Our results point toward a minimal contribution of carbonate weathering to the overall soil CO_2_ efflux. Source partitioning using intact soil cores in the laboratory indicated a ~2–3 % contribution of CO_2_ from dolomite weathering, whereas a contribution of abiotic CO_2_ from limestone weathering was not detectable at all. These estimates include a 7 ‰ equilibrium isotope effect on soil CO_2_ caused by DIC leaching (see Fig. 1b) and would be lower without accounting for the ^13^C depletion inferred by this process. We incubated intact soil cores in the laboratory to constrain the CO_2_ sources to heterotrophic CO_2_ and CO_2_ originating from carbonate weathering products. Although tree roots in the cores likely continued to respire at lower rates during the first incubation stage, substantial CO_2_ contribution from the cut-off roots was unlikely during the latter part of the long-term incubation (60 and 166 days, respectively). Autotrophic respiration can make up to 50 % of the total soil CO_2_ efflux at the dolomite site (Schindlbacher et al. 2009). If the weathering rate is similar as in the lab, then the contribution of weathering derived CO_2_ in the field would be about 1 %, given a 50 % contribution of autotrophic respiration to soil CO_2_ efflux. As autotrophic respiration increases the CO_2_ partial pressure by up to 2–3 times in the A/C and C-horizon (Table 3) it likely contributes to carbonate weathering during the growing season. Accordingly, our incubation data indicate a realistic abiotic CO_2_ contribution between 1 and 2 % to total soil CO_2_ efflux at the forest growing on dolomite bedrock. As mentioned in the method section, we found considerable amounts of carbonate in some A-horizon cores which were supposed to exhibit only heterotrophic soil respiration. Although we could not find any sign for significant abiotic CO_2_ production in A-horizon cores, a minimal abiotic CO_2_ release could have occurred. Accordingly, the abiotic contribution to the soil CO_2_ efflux would be slightly underestimated. Our estimates, however, hold some uncertainty in the reverse direction as well. As we compared A-horizon cores with whole soil profile cores, we implied that the heterotrophic respiration from all cores has the same isotopic signature. It turned out, however, that the δ^13^C values of the organic C became less negative with increasing soil depth (Table 1). If heterotrophic respiration in deeper soil layers was enriched in^13^C and contributed significantly to the soil CO_2_ efflux, then the preconditions of our mass balance (Eq. 3) had been violated and the abiotic contribution was overestimated. The difference in absolute CO_2_ efflux between A-horizon cores and whole soil profile cores (Fig. 3) suggests that most of the CO_2_ was produced in the A-horizon. However, a smaller part of the CO_2_ efflux from the whole soil profile cores originated from deeper layers and could have influenced (^13^C enriched) the isotope signature of the headspace CO_2_. Therefore the estimated 1–2 % CO_2_ from carbonate weathering should rather be seen as the upper limit for the abiotic contribution to the total soil CO_2_ efflux.

Our DIC data further constrain the potential contribution of abiotic CO_2_ from carbonate weathering. Drainage water DIC concentrations from the dolomite soil cores were around 30 mg L^−1^ in our lab experiment. Considering seepage of about 1000 mm year^−1^ at the dolomite site (Feichtinger et al. 2002), the annual export of DIC would be around ~0.3 t C ha^−1^ year^−1^. This value is within the range of DIC export in other similar forests in Europe (Kindler et al. 2011) and fits well with catchment data of the Inn river of which our dolomite site is part of. The weathering intensity in the Inn river catchment was estimated at 60 meq HCO_3_^−^ km^−2^ s^−1^ (corresponding to 0.23 t C ha^−1^ year^−1^) at a mean deep percolation rate of 750 mm (Szramek et al. 2007). Furthermore, our drainage water δ^13^C values were similar to those of other carbonate soils throughout Europe (~−15 ‰) (Kindler et al. 2011) suggesting that the majority of the DIC was of biogenic origin. In comparison to the annual soil respiration of ~7 t C ha^−1^ year^−1^ at the dolomite site (Schindlbacher et al. 2014), our roughly estimated DIC export of ~0.3 t C ha^−1^ year^−1^ makes less than 5 % of the annual soil CO_2_ efflux. Considered that only a minor fraction of these 5 % was abiotic (δ^13^C ~−15 ‰), and taking into account that most abiotic C is percolated, the contribution of abiotic C to the soil CO_2_ efflux must be minimal.

Minimal abiotic contribution to the soil CO_2_ efflux was supported by radiocarbon data which was assessed in a previous study at the dolomite site (Schindlbacher et al. 2012). Given that carbonate has a radiocarbon signature of −1000 ‰, even small amounts of CO_2_ released from this source have a strong impact on the radiocarbon signature of soil CO_2_ efflux. The radiocarbon signatures of the latter ranged between 21 and 76 ‰ (mean 54 ‰) at three sampling dates in the growing season of 2009, indicating that CO_2_ from dolomite weathering comprised on average not more than 1–1.5 % of the total soil CO_2_ efflux at this site.

Our incubation data suggest that the relative contribution of abiotic C is higher under drier conditions. We hypothesized (II) that wetter conditions foster carbonate dissolution and thereby increase the abiotic efflux-share whereas dryer conditions reduce carbonate weathering rates and the corresponding efflux. During our drying experiment, however, the following observations were made. Soil dried out very slowly because of low evaporation and lack of plant water use. During the first phase of the incubation only the litter layer and the very top-soil dried out whereas the larger part of the A-horizon as well as the deeper horizons remained moist. During the latter part of the incubation, the A-horizon had significantly dried out whereas the deeper horizons were still moist. Therefore, heterotrophic respiration in the SOM-rich upper soil layer was more affected by drying than carbonate weathering in the deeper and wetter soil horizons. Accordingly, the share of the abiotic contribution to the decreasing total soil CO_2_ efflux became larger. The pattern of soil moisture can be similar at the field site with driest litter layer and top-soil and comparatively wet sub-soil (Schindlbacher et al. 2012). Similar to our study, abiotic CO_2_ efflux in a Mediterranean shrubland commenced at low but steady rates, whereas decreasing soil moisture mostly affected the heterotrophic respiration of the dried out top-soil (Inglima et al. 2009).

Our lab data were generally in good agreement with the field measurements. Lab flux rates and soil CO_2_ concentrations were roughly half as high in the field during summer because of missing autotrophic respiration in the soil cores. The isotopic signature of the summertime field soil CO_2_ efflux and concentrations were also coherent with the lab data. An exception was the A-horizon where δ^13^C values of field soil CO_2_ were less negative than in the lab. This however was not surprising as the laboratory incubation was made under exclusion of atmospheric CO_2_ (δ^13^C values ~−8 ‰) which is considered to diffuse into the uppermost soil layers in the field. The seasonal variation in the isotopic signature of the soil CO_2_ efflux and CO_2_ concentrations in the field (Table 3) can have several reasons, one of them being variations in the contribution of abiotic CO_2_ from weathering. The dissolution rate of carbonate minerals is negatively related to temperature (Langmuir 1997) as well as the solubility of CO_2_ in water. Therefore, the relative contribution of abiotic CO_2_ to the soil CO_2_ efflux could be higher during the cold season. Indeed, we found less negative δ^13^C values of the soil CO_2_ during winter, which might be a hint in this direction. Similar patterns were observed by Carmi et al. (2013) who measured the δ^13^C values of the soil CO_2_ in a carbonate containing pine forest soil. Another reason for the less negative δ^13^C values of soil CO_2_ during winter could be higher mixing with atmospheric CO_2_, which occurs under lower soil respiration rates (Cerling 1984). The inverse pattern of δ^13^C values of soil CO_2_ in spring with less negative values in the deeper soil may be another indication for a potentially higher abiotic contribution. During spring, deeper soil layers are still cold but the CO_2_ partial pressure is already twice as high as during winter. Therefore enhanced carbonate weathering may have contributed to this atypical distribution of δ^13^C values throughout the soil profile. However, this is speculative because seasonal variations in autotrophic respiration and its isotopic signature could have influenced the isotopic signature of the field soil CO_2_ as well (Ekblad and Högberg 2001). The seasonal variation in the isotopic signature of the soil CO_2_ in the field suggests that our incubation based estimates of the abiotic CO_2_ efflux apply under growing season conditions whereas the relative abiotic contribution to the cold season soil CO_2_ efflux could be higher. As the wintertime soil CO_2_ efflux at our sites is in a range of ~10 % of the annual soil CO_2_ efflux (Schindlbacher et al. 2014), the effect on the annual C budget would, however, be small. At both of our field sites, the A-horizons showed small-scale variations in thickness (10–50 cm depth). CO_2_ efflux from soil with deep A-horizons is generally higher than from soil with shallow A-horizons (Schindlbacher, unpublished data). Accordingly, the relative contribution of abiotic CO_2_ will likely show high spatial variation in the field. Such small-scale variations in the abiotic contribution and hence in the isotopic signal of the soil CO_2_ efflux are relevant if natural abundance methods or radiocarbon studies are applied to forest soils on carbonate bedrock.

Our hypothesis (III) that the abiotic CO_2_ efflux is higher in the limestone soil could not be confirmed as well. While we already operated close to the detection limits of our experimental setup regarding the abiotic CO_2_ contribution from dolomite cores, we did not find evidence for an abiotic contribution to the soil CO_2_ efflux from the limestone cores. Due to the higher spatial variability of δ^13^C values of the soil CO_2_ efflux and the lower number of limestone cores, a minimal abiotic contribution to the soil CO_2_ efflux can, however, not be excluded. Generally, limestone dissolution is considered to occur at faster rates as dolomite dissolution (Chou et al. 1989; Morse and Arvidson 2002; Pokrovsky et al. 2005). Actual site specific weathering rates also depend on the degree of rock surface fracturing and probably also on microbial rock surface colonization (Davis et al. 2007). Site specific soil properties such as porosity and soil density might affect the transport and release of abiotic C which is produced predominately in the deeper soil layers and thereby also control the contribution to the total soil CO_2_ efflux. Therefore, a simple relationship between dissolution rates of the various carbonate bedrock and the abiotic soil CO_2_ efflux seems rather unlikely.

## Conclusions

Our lab incubation indicated only minimal abiotic contributions to the soil CO_2_ efflux at the dolomite site whereas an abiotic contribution was not detectable at the limestone site. This is in agreement with the radiocarbon signature of the soil CO_2_ efflux and with geochemical weathering rates and the expected downward leaching of most of the weathering products in moist temperate environments. Seasonal variations in the isotopic signature of the CO_2_ in the field soil indicate that our incubation data apply under growing season conditions whereas the abiotic flux component could be higher during winter. The overall low contribution of abiotic CO_2_ to the soil CO_2_ efflux may be negligible in most C budgeting and biotic source partitioning approaches where the abiotic efflux should largely fall within the uncertainty range of the methods applied. Our data suggest that the abiotic contribution to soil CO_2_ efflux varies in space, time, and with environmental conditions. Such variations would influence the isotopic signal of the soil CO_2_ efflux and therefore could bias isotopic studies if not accounted for.

## Electronic supplementary material

Fig. S1 Relationship between the isotopic signature of the soil air CO_2_ and the inorganic and organic C concentration of the A-horizon only cores (a, b, dolomite; c, d, limestone) (JPEG 83 kb)
