# Supplementary figures and images for: Contribution of carbonate weathering to the CO2 efflux from temperate forest soils
Source: Biogeochemistry. 2015 Apr 14;124(1-3):273–90. doi: 10.1007/s10533-015-0097-0 (PMC4512732; doi:10.1007/s10533-015-0097-0)

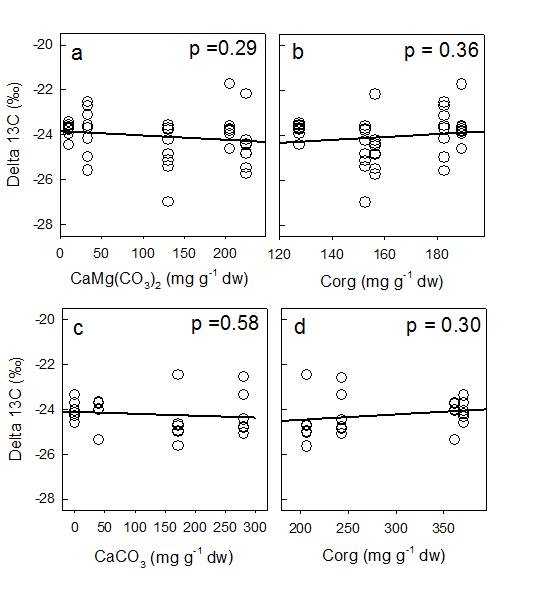

Supplement: Supplementary file 1 — Relationship between the isotopic signature of the soil air CO2 and the inorganic and organic C concentration of the A-horizon only cores (a, b, dolomite; c, d, limestone) (JPEG 83 kb) [file 10533_2015_97_MOESM1_ESM.jpg]
